# Antimicrobial Activities of European Propolis Collected from Various Geographic Origins Alone and in Combination with Antibiotics

**DOI:** 10.3390/medicines5010002

**Published:** 2018-01-03

**Authors:** Issam AL-Ani, Stefan Zimmermann, Jürgen Reichling, Michael Wink

**Affiliations:** 1 Institute of Pharmacy and Molecular Biotechnology, Heidelberg University, INF 364, 69120 Heidelberg, Germany; issam788@yahoo.com (I.A.-A.); juergen.reichling@urz.uni-heidelberg.de (J.R.); 2 College of Health and Medical Technology, Middle Technical University, Baghdad 10047, Iraq; 3 Department of Infectious Diseases, Medical Microbiology and Hygiene, Heidelberg University, INF 324, 69120 Heidelberg, Germany; Stefan.Zimmermann@med.uni-heidelberg.de

**Keywords:** propolis, chemical composition, antioxidant, antibiotics, synergy, MDR-bacteria

## Abstract

**Background:** Propolis consists of a complex mixture of resinous substances collected by honeybees from different plant sources. The objective of this study was to investigate the chemical composition, biological activities, and synergistic properties with antibiotics of propolis samples collected from various geographic origins (Germany, Ireland, and Czech Republic). **Methods:** The chemical composition of the propolis was analyzed by Gas Liquid Chromatography-Mass Spectrometry (GLC-MS) and High-performance liquid chromatography (HPLC). The minimal inhibitory concentration (MIC) and minimal bactericidal concentration (MBC) were evaluated by the standard broth microdilution method, while synergistic interactions were assessed by checkerboard dilution and time-kill curve assays. **Results:** HPLC and GLC-MS analyses revealed that ethanol extract of propolis (EEP) and water extracts of propolis (WEP) contained more than 100 different phytochemicals. The most abundant compounds were aromatic alcohols, aromatic acids, cinnamic acid and its esters, fatty acids, and flavanone (chrysin). Czech propolis showed the highest phenolic content (129.83 ± 5.9 mg CAE/g) followed by Irish propolis and German propolis. Furthermore, Irish propolis exhibited the highest value of total flavonoid content (2.86 ± 0.2 mg QE/g) and antioxidant activity (IC50 = 26.45 µg/mL). All propolis samples showed moderate antibacterial effect against Gram-positive microorganisms with MIC ranging from 0.08 mg/mL to 2.5 mg/mL. Moreover, EEP exhibited moderate activity against Gram-negative bacteria with MIC between 0.6 mg/mL to 5 mg/mL. In addition, EEP displayed moderate antifungal activity (MIC values between 0.6–2.5 mg/mL). The results obtained from time kill-kinetic assay and checkerboard dilution test of two-drug combinations between EEP and antibiotics such as vancomycin, oxacillin, and levofloxacin indicate mainly synergistic interactions against drug-resistant microbial pathogens including MRSA and VRE. **Conclusions:** The propolis extract synergistically enhanced the efficacy of antibiotics, especially those acting on cell wall synthesis (vancomycin and oxacillin) against drug-resistant microorganisms.

## 1. Introduction

Propolis (bee glue) is a sticky resinous substance produced by honey bees from different plant sources such as leaves, flowers, and bud exudates, modified by bee secretions and wax [1]. The word propolis “The Greek pro = in defense or for, and polis = city” reflect its significance to honey bees, since they employ it to smooth out internal walls, as well as to defend the colony against infections [2]. Propolis contain natural mixtures of different secondary metabolites that are responsible for various bioactivity such as antibacterial, anti-angiogenic, antiulcer, anti-inflammatory, antioxidant, and anti-viral activities [3].

The typical raw propolis is consists of 45–55% plant resin, 25–35% wax, 5–10% essential and aromatic oil, 5% pollen and 5% other natural products [4]. Moreover, propolis contains various kinds of other secondary plant metabolites, which differ in concentrations depending on season, geographic origins of the collection and the proximity of a beehive to particular plant sources.

The major ingredients of propolis collected from zones of Europe Asia, North America etc. are characterized by many phenolic ingredients including flavonoids, aromatic acids and their esters, frequently gathered by honey bees from poplar buds (*Populus* spp.). These compounds are predominant compounds in poplar buds, and known to exhibit several biological and pharmacological properties. Polyprenylated benzophenones and various diterpenes were the main compounds found in tropical propolis collected from tropical zones such as Brazil [5]. 

Several groups of researchers documented that all types of propolis have antibacterial properties. Veiga et al. [6] reported that poplar propolis has antibacterial effect against both Gram-positive and Gram-negative microorganisms including multidrug-resistant bacteria such as Methicillin-resistant *Staphylococcus aureus* (MRSA), while Yildirim et al. [7] investigated the effect of Turkish propolis against tuberculosis and they found that water extract of propolis has anti-tuberculosis activity against different types of mycobacteria. In addition, many studies have documented the remarkable action of propolis against many types of microorganisms including yeasts, viruses, bacteria, and parasites [8]. Besides the bioactivities and pharmacological properties of propolis, a number of studies indicate that propolis has no toxicity and no side effects in animal models or humans [9]. 

The goal of this study was to investigate and compare the chemical composition, biological activities of propolis samples collected from various geographic areas (Germany, Ireland, and Czech Republic). In addition, synergistic properties between the ethanol extract of propolis (EEP) and the antibiotics vancomycin, oxacillin, and levofloxacin could be demonstrated in MDR bacteria, which might be important for future uses of propolis in the clinic to combat antibiotic resistant pathogens.

## 2. Materials and Methods

### 2.1. Chemicals and Drugs

All chemicals used in this study were purchased from Sigma–Aldrich (Steinheim, Germany). DMSO came from Grüssing GmbH (Filsum, Germany). The standard drugs including streptomycin and levofloxacin were obtained from Applichem (Darmstadt, Germany), vancomycin from Carl Roth^®^ (Karlsruhe, Germany), oxacillin from Sigma–Aldrich (Steinheim, Germany), and nystatin from Cellpharm (Hannover, Germany).

### 2.2. Ethanol Extract Preparation of Propolis

Propolis was collected from beekeepers in Germany, Ireland, and the Czech Republic. Crude propolis was ground and extracted according to Morsy et al. [10] with slight modifications. Briefly, the samples were ground into very fine powder by a blender and 10 g of the propolis mixed in 100 mL of 70% ethanol and shaken at 37 °C for 24 h. Then, centrifuged at 26,000× *g* for 30 min and filtrated by filter paper (Whatman No. 4). Rotary evaporator was used at 50 °C with low pressure to evaporated the remaining of ethanol. The sample was kept at 4 °C in the dark until use. For antimicrobial experiments EEP was diluted in dimethyl sulfoxide (DMSO) with in a final DMSO concentration lower than 1%, which is non-lethal for microorganisms.

### 2.3. Water Extract Preparation of Propolis

Aqueous German propolis extract was prepared as designated by Miguel et al. [10] with slight modification. 10 g of the dried powder of propolis was crushed into a very fine powder in a blender, dissolved in 20 mL of sterile water and kept at 60 °C for 7 h. The suspensions were separated by centrifuge at 28,000× *g* for 30 min and then filtrated by filter paper (Whatman No. 1). Rotary evaporator was used at 65 °C with low pressure to dispose of excess water and extract (WEP) was kept under 4 °C in the dark until testing.

### 2.4. Gas Liquid Chromatography/Mass Spectrometry (GLC/MS) Analysis of Propolis

GLC/MS was performed on a Hewlett-Packard gas chromatograph GC 5890 II “Hewlett-Packard 93 GmbH, Bad Homburg, Germany” equipped with a 25 m DB-5 capillary column with a (5% phenyl)-polymethyl siloxane stationary phase a film thickness of 0.25 µm. Propolis extracts were dissolved in methanol, and 2 µL of sample was injected with a split mode (split ratio 1:100) with the carrier gas helium at a flow rate of 2 mL/min. The capillary column was coupled to a quadrupole mass spectrometer (Finnigan SSQ, Bremen, Germany) and optimized instrumental parameters were as follows: Injector temperature (250 °C), head pressure (15 hPa), and transfer line heater (280 °C). The mass spectra was noted according to the following recommendations : Scan range (Routine): *m*/*z* 40–600, scan time: (Routine) 1 s, emission current: 100 µA, electron energy: 70 eV, source temperature: 175 °C, filament delay time: (Routine) 3 min; with Xcalibur homepage version 1.3 (Thermo Finnigan, San Jose, CA, USA) data system [11,12].

### 2.5. High-Performance Liquid Chromatography (HPLC) Analysis of Propolis

Approximately 500 µg of propolis extracts were dissolved in 2 mL of methanol and filtered with a 0.45 μm membrane filter, centrifuged for 10 min at 13,000 rpm. Then 20 µL supernatants were injected with an auto-injector into the HPLC system (Beckman Gold HPLC, Burnsville, MN, USA) with a solvent module (125P, PDA detector 168) and a LiChroCART RP18 column (5 µm, 250 × 4 mm, Merck, Kenilworth, NJ, USA) using as mobile phase H_2_O with 1% formic acid (solvent A) and acetonitrile with 1% formic acid (solvent B). The elution carried out with a linear gradient at a flow rate of 1 mL/min. The detection was monitored at 300 nm and Xcalibur 2.0 software (Thermo scientific, Waltham, MA, USA) was used for analysis.

Authentic standard compounds such as chrysin, pinocembrin, and galangin were commercially obtained from Gehrlicher Pharmaceutical extracts (Eurasburg, Germany), cinnamic acid from Carl Roth, (Karlsruhe, Germany), caffeic acid, and *p*-coumaric acid from Sigma–Aldrich (Steinheim, Germany) [11].

### 2.6. Antioxidant Activity of Propolis 

Antioxidant activity is based on the decrease in the absorbance when the diphenyl-picrylhydrazyl radical (DPPH•) is reduced at 517 nm. This assay depends on the fact that the purple color of the DPPH• radicals is bleached to a yellow color in presence of any molecule that can donate an electron or proton. Equal volumes of propolis extract were mixed with 0.2 mM methanol solution of DPPH• and incubation in the dark for at 37 °C for 30 min [13]. After incubation, the absorbances of the mixtures were measurement against a blank at 517 nm using a Tecan^®^ Safire II Reader. DPPH• with methanol was employ as control, while ascorbic acid was employ as reference in comparing to the propolis extract. The efficacy of antioxidant in the samples were expressed in percentage of DPPH• reduction and was calculated using the formula below:

Inhibition (%) = 100 × [A517 (control) − A517 (sample)/A517 (control)]

### 2.7. Determination of Total Phenolic Content (TPC) of Propolis

The total phenolic content was determined using the method of Folin–Ciocalteu as designated previously by Köksal et al. [13]. Propolis sample with concentrations of 10 mg/mL was prepared to use in the analysis. 20 µL of propolis sample extract and the standard solution (caffeic acid) with concentration range 0–200 µg/mL was pipetted into a round bottom 96-well plate and 100 µL of Folin-Ciocalteu solution was added to the well and left for 5 min at 37 °C. Then, 80 µL of Na_2_CO_3_ solution was added and mixed well. The plate was incubated at 37 °C for two hours, the absorbances of the reactions were recorded at 760 nm with Biochrom Asys UVM 340 microplate reader against a methanol as blank. Based on the measured absorbance of the caffeic acid and the calibration curve was constructed. The contents of phenolic in the extracts were expressed in terms of caffeic acid equivalent (CAE). The total phenolic content was expressed in mg of caffeic acid equivalents (CAE)/g of extract.

### 2.8. Determination of Total Flavonoid Content (TFC)

The aluminum colorimetric technique was used for evaluation of the total flavonoid content in propolis extracts as recommended by Köksal et al. [13]. Briefly, 150 μL (0.4 mg/mL) of propolis extract were mixed with 2% (*w*/*w*) AlCl_3_ (100 μL) in a 96-well microplate. Then, incubated at 37 °C for 30 min and record the absorbance at 415 nm with a Biochrom Asys UVM 340 microplate reader against a blank (a sample without aluminum chloride). Quercetin was used as the standard and the curve of calibration was plotted versus standard curve of quercetin and the data was expressed as quercetin equivalent (QE) per g of propolis extract.

### 2.9. Microorganisms and Culture Media

A total of 32 reference strains (Gram-positive bacteria, Gram-negative bacteria, and fungi) from American Type Culture Collection (ATCC) were used in this study and are listed in Tables 2 and 3. In addition, one strain of Methicillin-resistant *Staphylococcus aureus* (MRSA), one strain of Vancomycin-resistant enterococci (VRE), two strains of Gram-negative bacteria and four strains of fungi isolated from clinical sources were included in this study. All microorganisms were supplied by the Medical Microbiology Lab., Hygiene Institute, Heidelberg University, Germany.

All bacterial strains were cultivated on Columbia Agar supplemented with 5% sheep blood (Becton Dickinson, Heidelberg, Germany) and cation-adjusted Muller-Hinton broth (CAMHB) (Fluka, Buchs, Switzerland) except VRE and streptococci, for which Brain Heart Infusion (BHI) (Merck, Darmstadt, Germany) was used. Sabouraud Dextrose broth (SDB) (Merck, Darmstadt, Germany) and CHROMagar Candida medium (Becton Dickinson, Heidelberg, Germany) were employed for the cultivation of fungi.

### 2.10. Evaluation of Minimum Inhibitory Concentrations (Mics) and Minimal Bactericidal Concentrations (MBC) of Propolis

The MICs and MBC activity of propolis extract or antimicrobial agents were carried out as recommended by Clinical and Laboratories Standards Institute (CLSI, Wayne, PA, USA). Briefly, two on three of bacterial or fungal colonies form agar media were dissolved in test tubes contain normal saline to get inoculum suspensions with concentration of 1 × 10^6^ bacteria and 1 × 10^5^ yeast cells. Then, inoculum suspensions distributed in to a 96-well microtiter plate containing two-fold serial dilution of the propolis samples. The MIC value was reported as the lowest concentration of propolis which inhibited bacterial or fungal growth after incubation at optimal temperature. The MBC values of propolis were evaluated by sub-culturing about 5–10 μL of wells with concentration equal or higher than MIC on blood agar plate for bacteria and sabouraud dextrose agar for fungi [14]. Antibiotics such as streptomycin or vancomycin were used as positive controls.

### 2.11. Checkerboard Dilution

The broth microdilution checkerboard technique was employed to study the synergistic effect between propolis extract with selected antibiotics. Briefly, two-fold serial dilution was used in the distribution of propolis extract and selected antibiotics in a 96-well microtiter plate with sub-MIC concentration. Then, a 100 μL of inoculum equal to 1 × 10^6^ colony-forming unit (CFU)/mL from bacteria was distributed in to each well and incubated for 24 h at 37 °C. Fractional inhibitory concentration index (FICI) was computed by the accompanying equation:

“FICI = (MIC of antimicrobial agent A in combination/MIC of antimicrobial agent A alone) + (MIC of antimicrobial agent B in combination/MIC of antimicrobial agent B alone)”.

The FIC index was considered as a synergistic when it was ≤0.5, as additive when it was >0.5–1, indifferent when it was ≥1–4.0, and antagonistic when it was >4 [15].

### 2.12. Time-Kill Assays

Time-kill kinetics assay was performed according to recommended of CLSI. Briefly, tubes containing cation-adjusted Mueller-Hinton broth with sub-MIC concentration of antibiotics and propolis incubated at 37 °C with 1 × 10^6^ CFU/mL of bacterial suspensions. Then, aliquots of 100 μL removed from all tube after incubation period at (0, 2, 4, 6, and 24 h) and ten-fold serial dilution was prepared with normal saline and aliquots of 10 μL from dilutions was plated by sterile wound swab on to blood agar and incubated at optimal temperature for 18–24 h. The number of bacterial colonies were calculated on blood ager to count of CFU/mL. The combinations decrease of CFU/mL by ≥2 log_10_ considered as synergy [14].

### 2.13. Data Analysis

All experiments in this study were completed in triplicate and introduced as mean ± SD (standard deviation). GraphPad Prism^®^ software was employed for statistical analysis using One-way ANOVA or student’s *t*-test with Bonferroni method. *p*-value less than 0.05 accepted as significant.

## 3. Results

### 3.1. Propolis Extracts Analysis

**Figure 1:**
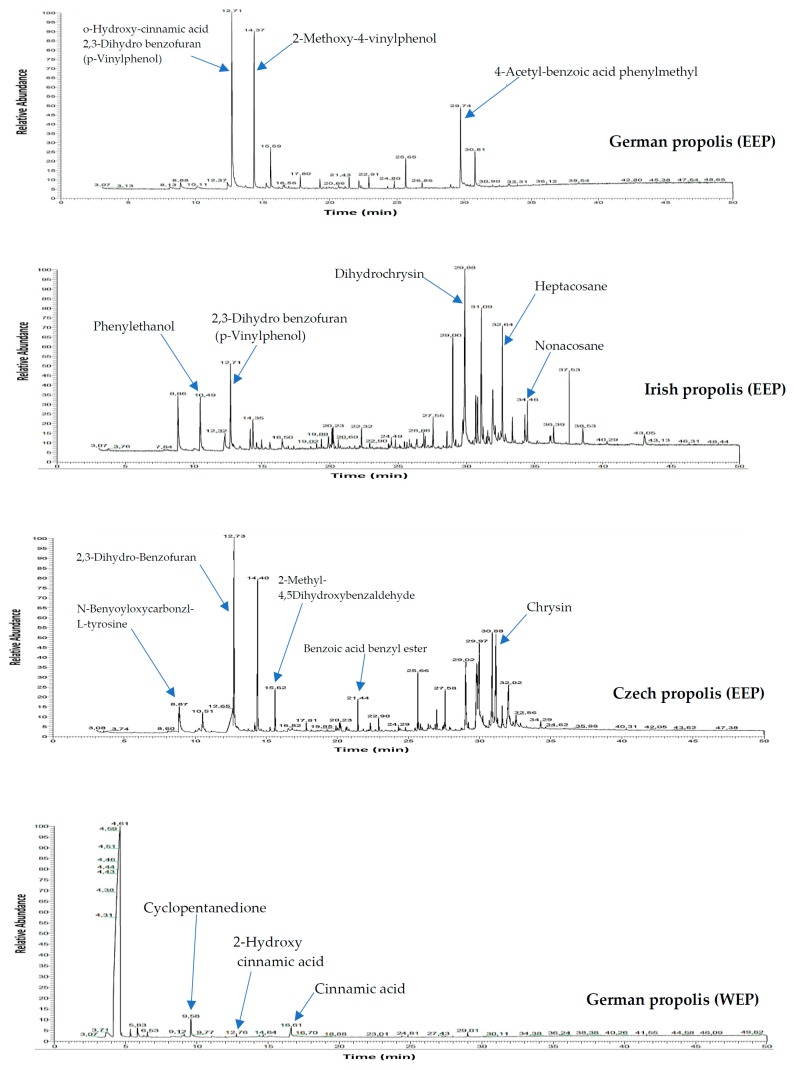
GLC-MS profile of ethanol and water extracts of propolis.

Crude propolis collected from different geographical regions in Europe including Germany, Ireland, and Czech Republic were extracted with ethanol or water to study their chemical composition, antioxidant properties, and antimicrobial activity alone and in combination with antibiotics. Ethanol and aqueous extracts of propolis were first investigated by GLC-MS and HPLC to detect the major chemical patterns. As shown in Figure 1 the composition of European propolis show various chemical compounds depending on their geographical origin. Data analysis of GLC-MS and HPLC provided evidence for more than 100 compounds in the ethanol and aqueous extracts (Table 1).

The following compounds were identified as plentiful in the analysed samples: Benzoic acid, benzoic acid benzyl ester, cinnamyl alcohol, benzyl cinnamate, cinnamyl cinnamate, cinnamic acid ethyl ester, eudesmol, phenylethanol, cinnamic acid, 4-vinyl-2-methoxy-phenol, 4-vinyl-methoxy-phenol, 4-hydroxybenzaldehyde, alpha-bisabolol, myristic acid ethyl ester, hexadecanoic acid, benzyl alcohol, stearic acid, 2′,6′-dihydroxy-4′-methoxy chalcon, and dihydrochrysin.

German propolis was characterized by several acids including benzoic acid, cinnamic acid, 4-methoxyphenyl propanoic acid, dodecanoic acid, myristic acid, salicylic acid, and hexadecanoic acid. Irish propolis contained many flavonoids such as chrysin, galangin and pinocembrin as well as significant amounts of caffeic acid, nonacosane, pentacosane, heptacosane, eudesmol, guaiol, and alpha-bisabolol. On the other hand, phenyl carboxylic acids (caffeic acid, cinnamic acid, *p*-coumaric acid and, benzoic acid) and flavonoids (chrysin, galangin, and pinocembrin) were predominant compounds in Czech propolis.

In contrast, aqueous extracts of propolis had a few compounds including 2-furanmethanol, cyclopentanedione, 2-hydroxy cinnamic acid, 2-methoxy-4-vinylphenol, 4-vinyl-2-methoxy-phenol, hexadecanoic acid, stearic acid ethyl ester, 2′,6′-dihydroxy-4′-methoxy-chalcone, and cinnamic acid.

### 3.2. Total Phenolic and Flavonoid Content of Propolis

**Figure 2:**
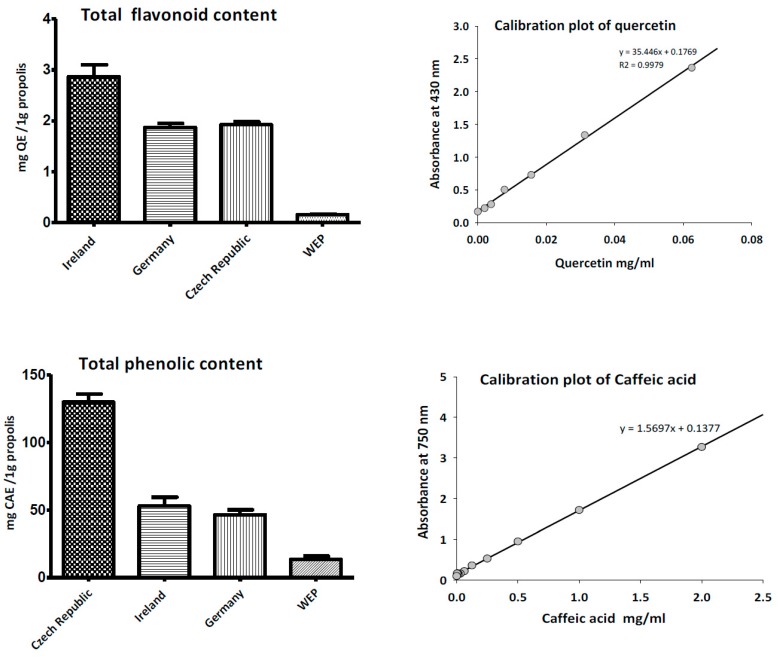
Total phenolic and flavonoid content of ethanol and water extracts of propolis.

The total phenolic content of propolis EEP was determined according the Folin–Ciocalteu assay and represented in terms of caffeic acid equivalent (CAE), while total flavonoids contents were estimated by the aluminium colorimetric technique and expressed as quercetin equivalent (QE). Figure 2 illustrates the propolis sample collected from Czech Republic that had the highest phenolic content (129.83 ± 5.9 mg CAE/g), followed by Irish propolis (52.81 ± 4.3 mg CAE/g), and German propolis (46.45 ± 3.1 mg CAE/g). On the other hand, Irish propolis had the highest content of total flavonoids contents (2.86 ± 0.2 mg QE/g) in comparison with other propolis samples collected from other countries. The aqueous propolis extract exhibited minimum flavonoid content (0.11 ± 0.01 mg QE/g).

### 3.3. Antioxidant Activity of Propolis

**Figure 3:**
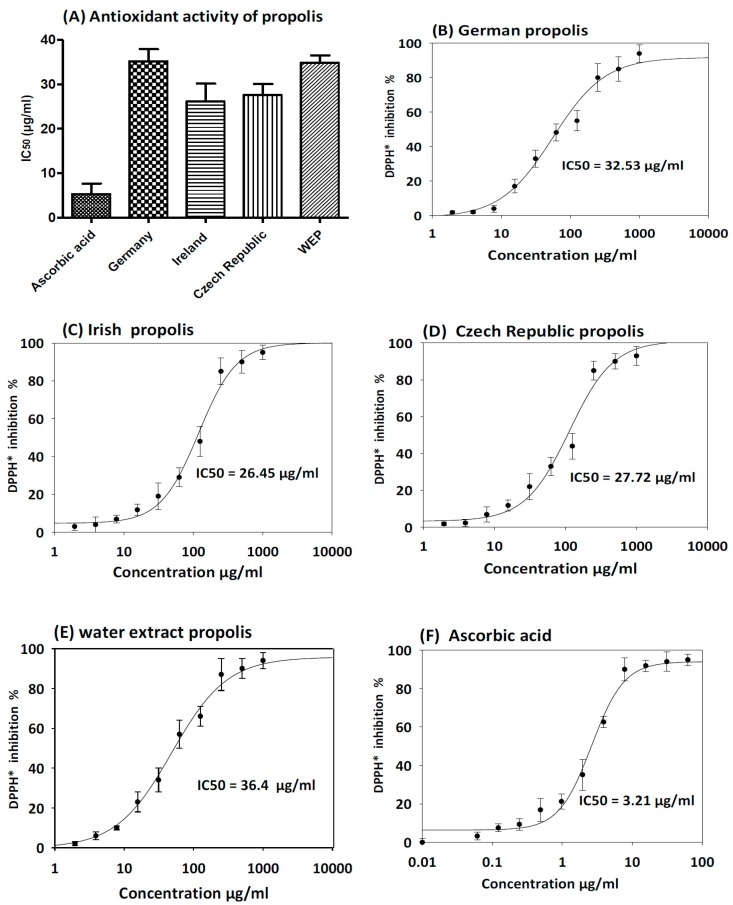
DPPH free-radical scavenging activity of propolis extracts. The results are expressed as mean ± SD. (**A**) Antioxidant activity of ethanol and water extract of propolis compare to ascorbic acid as control. (**B**) Antioxidant activity curve of German EEP. (**C**) Antioxidant activity curve of Irish EEP. (**D**) Antioxidant activity curve of Czech Republic EEP. (**E**) Antioxidant activity curve of WEP. (**F**) Antioxidant of ascorbic acid standard curve.

The antioxidant activity of ethanol and water extract of propolis was evaluated using the DPPH method with ascorbic acid as a control. As shown in Figure 3, all ethanol extracts showed free radical scavenging activity with IC_50_ ranging between 26.45 ± 3.4 µg/mL and 36.40 ± 3.2 µg/mL. Propolis samples collected from Ireland and Czech Republic demonstrated the highest free radical scavenging activity with IC_50_ 26.45 ± 3.8 µg/mL and 27.72 ± 5.2 µg/mL respectively. As expected from the analyses of total phenolics and flavonoids, aqueous extract of propolis displayed moderate antioxidant activity with IC_50_ 36.40 ± 3.2 µg/mL.

### 3.4. Antimicrobial Activity of Propolis

All propolis extracts evaluated in this study showed antibacterial effect against Gram-positive bacterial pathogens with MIC ranging from 0.08 mg/mL to 5 mg/mL (Table 2). Irish propolis showed remarkable bactericidal effect against Gram-positive microorganisms followed by Czech, and German. *Bacillus subtilis* and *Streptococcus pyogenes* were highly sensitive Gram-positive microorganisms to EEP. Moreover, both types of propolis displayed a moderate anti-MRSA and anti-VRE efficacy against both reference and clinical isolates strains with MIC between 0.3 mg/mL to 2.5 mg/mL.

Most ethanol extracts of propolis exhibited moderate efficacy against Gram-negative microorganisms with MIC between 0.6 mg/mL to 5 mg/mL. *P. aeruginosa* display high resistant Gram-negative bacteria towards propolis. On the contrary, aqueous extract of propolis showed low bactericidal activity against Gram-negative bacteria (MIC ranging from 1.2 mg/mL to 5 mg/mL) (Table 3). Furthermore, EEP and WEP exhibited moderate effect against human respiratory bacterial pathogens including positive β-lactamase production *Haemophilus influenzae*, and *Streptococcus pneumoniae* (MIC between 0.6 mg/mL to 5 mg/mL).

All propolis samples exhibited antifungal activity against reference and clinical isolatesd strain. EEP from Ireland and Czech demonstrated excellent fungicidal effect with minimum fungicidal concentration (MFC) between 0.1 mg/mL and 2.5 mg/mL, while propolis from other origins showed mostly fungistatic activity (MIC values between 0.6–5 mg/mL). *Candida glabrata*, *Candida parapsilosis*, and *Candida tropicalis* were the most sensitive Candida species (Table 4).

### 3.5. Evaluating Synergistic Interactions of Propolis Extracts with Antibiotics in Checkerboard Assays

As indicated in in the Table 5, the results of checkerboard dilution of two-drug combinations between ethanol or aqueous extracts of propolis with antibiotics (vancomycin, oxacillin, and levofloxacin) demonstrated synergistic interaction against all tested microorganisms. The data of two-drug combinations were represented as isobolograms.

Two-drug combinations of EEP collected from Ireland either with vancomycin or oxacillin against MRSA and VRE exhibited synergistic FIC index values of 0.38 and 0.5, respectively. Synergism also was detected in the combination of EEP and vancomycin against *Streptococcus pyogenes* (FICI = 0.5). In addition, combination of EEP and levofloxacin revealed synergistic interaction against fastidious human respiratory bacterial pathogens including *Streptococcus pneumoniae* and *Haemophilus influenzae* (FICI = 0.5).

### 3.6. Time Kill Assays

**Figure 4:**
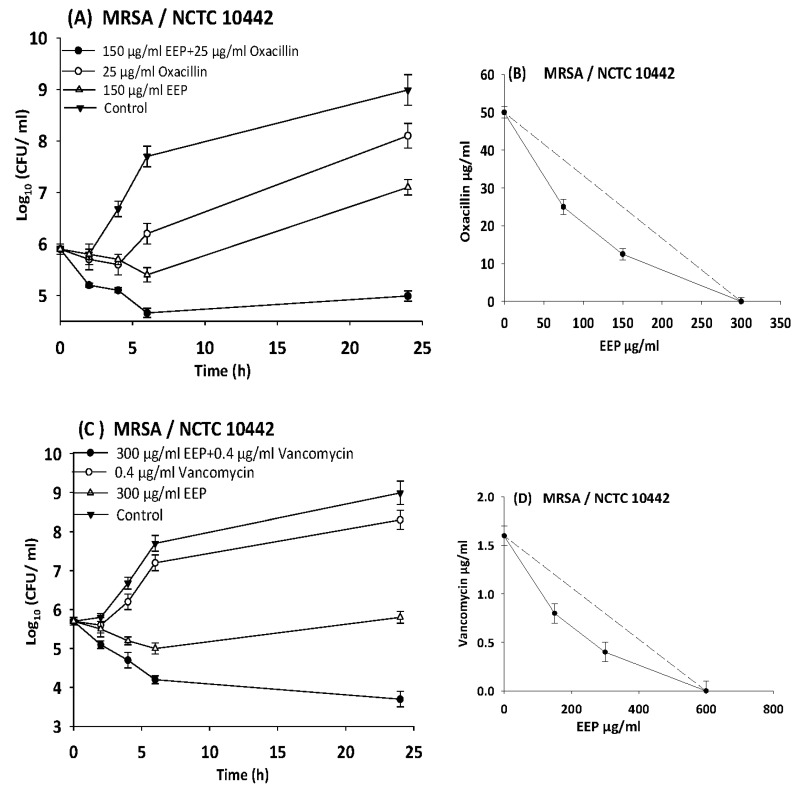
Time-kill curves and isobologram analyses shows synergistic interactions between ethanol extract of propolis and antibiotics against MRSA NCTC 10442. (**A**) Time-kill curves of combination between EEP and oxacillin. (**B**) Isobologram of combination between EEP and oxacillin. (**C**) Time-kill curves of combination between EEP and vancomycin. (**D**) Isobologram of combination between EEP and vancomycin.

**Figure 5:**
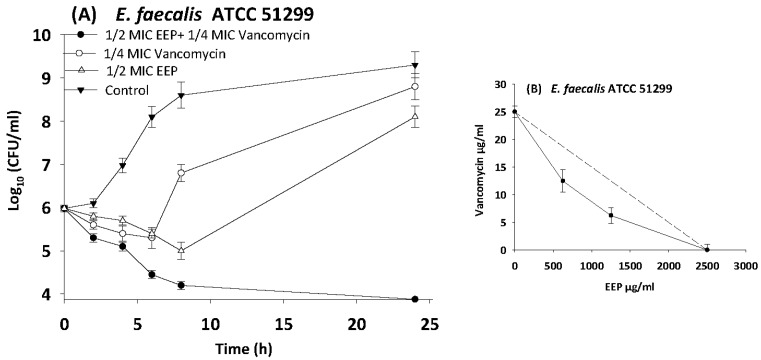
Time-kill curves and isobologram analyses shows synergistic interactions between ethanol extract of propolis and vancomycin against VRE ATCC 512999. (**A**) Time-kill curves of combination between EEP and vancomycin. (**B**) Isobologram of combination between EEP and vancomycin.

**Figure 6:**
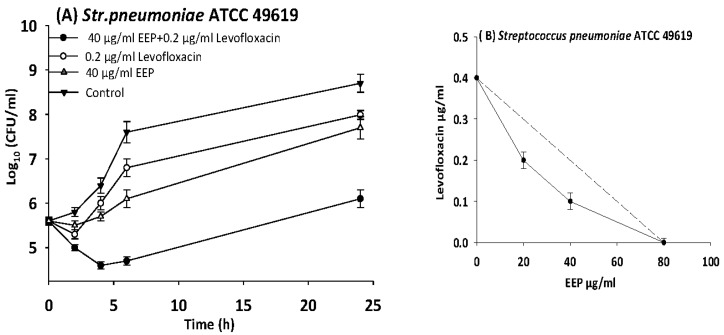
Time-kill curves and isobologram analyses shows synergistic interactions between ethanol extract of propolis and levofloxacin against *Streptococcus pneumoniae*. (**A**) Time-kill curves of combination between EEP and levofloxacin. (**B**) Isobologram of combination between EEP and levofloxacin.

**Figure 7:**
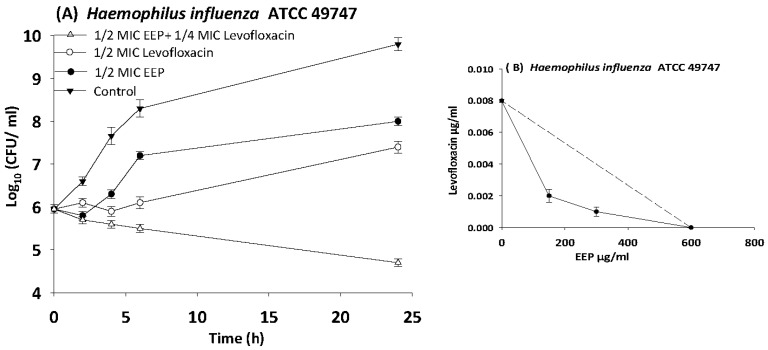
Time-kill curves and isobologram analyses shows synergistic interaction between ethanol extract of propolis and levofloxacin against *Haemophilus influenzae*. (**A**) Time-kill curves of combination between EEP and levofloxacin. (**B**) Isobologram of combination between EEP and levofloxacin.

**Figure 8:**
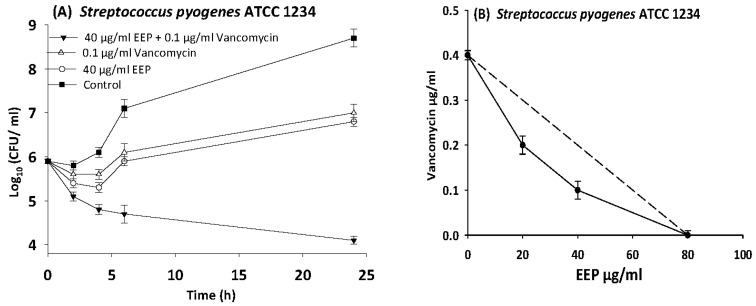
Time-kill curves and isobologram analyses shows synergistic interaction between ethanol extract of propolis and vancomycin against *Streptococcus pyogenes*. (**A**) Time-kill curves of combination between EEP and vancomycin. (**B**) Isobologram of combination between EEP and vancomycin.

The results of time kill-kinetic assay of two-drug combinations between EEP and WEP with antibiotics confirmed the results obtained from checkerboard assays. The two-drug combination of sub-MIC (½ MIC) of EEP with sub-MIC (¼ MIC) of vancomycin against *Streptococcus pyogenes*, VRE ATCC 51299, and MRSA NCTC 10442 display synergistic interactions with bacteriostatic effects and more than 3log_10_ reduction in colony count after overnight incubation in comparison with vancomycin as reference drugs. Additionally, two-drug combination of ½ MIC EEP with ½ MIC oxacillin revealed a synergistic interaction against MRSA NCTC 10442 with 3log_10_ decrease in colony counts after overnight incubation h in comparison with oxacillin as the most active single substance. Furthermore, synergistic interactions were noted when combining ½ MIC EEP with ½ MIC levofloxacin against fastidious bacteria (*H. influenzae* and *Str. pneumoniae*) with a significantly 3log_10_ reduction in colony counts after overnight incubation as compared with levofloxacin as the strongest single agent (Figure 4, Figure 5, Figure 6, Figure 7 and Figure 8).

## 4. Discussion

The complex chemical composition of propolis relying upon the plant origin, geographical location, and the collection seasons. Our propolis contained more than 100 substances in different concentrations such as phenolics, flavonoids and alkaloids that are responsible for its biological and pharmaceutical properties [16]. Several studies concluded that Asian, African, and European propolis contains predominantly phenolics and flavonoids such as naringenin, galangin, pinocembrin, apigenin, pinobanksin, quercetin, cinnamic acid and its esters, kaempferol, chrysin, cinnamyl caffeate, cinnamylidene acetic acid, caffeic acid, *p*-coumaric acid, aromatic acids and their esters [17,18]. Our GLC/MS and HPLC analysis results corroborate these findings. Chrysin, galangin, pinocembrin, *p*-coumaric acid and caffeic acid were the main components in ethanol extract of our propolis samples. 

In this investigation, we investigated the antioxidant activity of EEP and WEP from various geographic origins. According to our DPPH assay, Irish and Czech propolis had the strongest antioxidant activity with IC_50_ 26.45 ± 3.8 µg/mL and 27.72 ± 5.2 µg/mL respectively. The strong antioxidant activities were apparently related with the total phenolic and flavonoid content in the samples. On the contrary, WEP propolis showed the weakest antioxidant activity (IC_50_ = 36.40 ± 3.2 µg/mL) and exhibited the lowest amount of total phenolic and flavonoid content. These data were in agreement with those of Danert et al. [19] and Socha et al. [20] in terms of phenolic and flavonoids contents and radical scavenging activity.

Numerous studies have demonstrated that propolis possess a marked antibacterial, antiviral, and moderate antifungal activity [21,22,23]. The results presented by Seidel et al. [24] documented that propolis of North American, South American and European origins had MIC ranging from 0.125 to >0.5 mg/mL, while samples of African and Asian origin had MIC ranging from 0.08 to >0.5 mg/mL. The data of this study displayed that propolis exerts had bactericidal effects against Gram-positive microorganisms with MIC range from 0.04 to 1.2 mg/mL. However, it had a bacteriostatic effect against Gram-negative microorganisms, with MIC ranges from 0.6 to >5 mg/mL. These antimicrobial activity results are in line with other findings stating that Gram positive are sensitive to low propolis concentration and Gram-negative bacteria only inhibited with higher propolis dose [16]. This difference could be attributed to variable cell wall and membrane structure of the corresponding organisms. The bioactivities of propolis is not directly related to concentration of the biological active substances such as phenolic acid esters and flavonoids (pinocembrin and galangin), but a synergistic activity between these various active ingredients is believed to be a main factor in achieving the complex antimicrobial activity of propolis [25].

It is documented that propolis has different antibacterial mechanisms, including inhibition of cell division, collapsing microbial cytoplasm cell membranes and cell walls, inhibition of bacterial motility, enzyme inactivation, bacteriolysis, and protein synthesis inhibition [26,27]. The polyphenols of propolis will interact with many microbial proteins by forming hydrogen and ionic bonds, thus altering their three-dimensional (3D) structure of a protein and as a consequence their functionality [28,29]. These multi-target effects encouraged researchers to employ propolis to overcome drug resistance in microorganisms by combining propolis with antibiotics. Synergistic properties between EEP and antibiotics have been described by Orsi et al. [30]; they described a synergism between EEP and antimicrobial substances targeting microbial ribosomes (neomycin), but not with antimicrobial effective the biosynthesis of folic acid or DNA (ciprofloxacin and norfloxacin) nor those inhibiting metabolic pathways (cotrimoxazole). Wojtyczka et al. [31] mentioned synergistic interaction between EEP and antibiotics (chloramphenicol, gentamicin, netilmicin, tetracycline, tobramycin, and linezolid) interfering with bacterial protein biosynthesis against drug-sensitive and drug-resistant bacterial pathogens.

In the present study, we observed synergism between EEP and antibiotics that inhibit bacterial cell wall synthesis (vancomycin and oxacillin) against *Streptococcus pyogenes*, MRSA NCTC 10442, and VRE ATCC 51299 with more than 3.5 log_10_ reduction in colony count after overnight incubation.

In conclusion, we could confirm broad-spectrum bioactivities of ethanol extracts from propolis, whose major constituents were polyphenols and flavonoids. The propolis extracts synergistically enhanced the efficacy of antibiotics, especially those acting on cell wall synthesis (vancomycin and oxacillin). Further investigations are wanted to study the complex molecular mechanisms responsible for these synergistic interactions in order to develop new drug combinations for treatment multi-drug resistant bacterial infections.

## Figures and Tables

**Table 1:** Determination of the chemical composition of propolis EEP and WEP by GLC/MS and HPLC.

No.	Compounds	R_t_ (min) GLC-MS		EEP		WEP
			Germany	Ireland	Czech	Germany
1	2-Furanmethanol	5.33	−	−	−	+
2	Cyclopentanedione	6.53	−	−	−	+
3	benzyl alcohol	6.86	+	+	−	−
4	Phenol	8.12	−	−	−	−
5	*N*-Benyoyloxycarbonzl-l-tyrosine	8.87	−	−	+	−
6	2-Methoxyphenolacetat	10.15	+	−	−	−
7	1,2,3-Propanetriol monoacetat	10.27	−	−	+	−
8	Phenylethanol	10.49	−	+	−	−
9	Phenylethanol	10.51	−	−	+	−
10	Benzoic acid ethyl ester	11.7	−	−	−	−
11	Benzoic Acid	12.32	+	+	+	−
12	*O*-Hydroxy-cinnamic acid	12.71	+	−	−	−
13	2,3-Dihydro benzofuran (p-Vinylphenol)	12.71	−	+	−	−
14	2,3-Dihydro-Benzofuran	12.73	−	−	+	−
15	2-Hydroxy cinnamic acid	12.76	−	−	−	+
16	Cinnamyl alcohol	14.16	−	+	+	−
17	2-Methoxy-4-vinylphenol	14.35	+	+	+	+
18	4-Vinyl-2-Methoxy-phenol	14.52	−	+	−	+
19	Hydrocinnamic acid ethyl ester	14.98	−	+	−	−
20	4-Hydroxybenzaldehyde	15.26	+	−	+	−
21	4,5-Dihydroxy-2-methyl-benzaldehyde	15.59	+	−	−	−
22	3-Hydroxy-4-methoxy-benzaldehyde	15.6	−	+	−	−
23	2-Methyl-4,5Dihydroxybenzaldehyde	15.62	−	−	+	−
24	4-Hydroxybenzaldehyde	16.5	−	+	−	−
25	Cinnamic acid	16.5	−	−	−	+
26	4-Hydroxy-acetophenone	16.6	+	−	−	−
27	4-Propyl-guajacol (=2-Methoxy-4-propyl-phenol)	16.64	+	−	−	−
28	Cinnamic acid ethyl ester	16.94	+	+	−	−
29	p-Methoxyphenyl-2-Butanone	17.35	−	+	−	−
30	4-Hydroxy-3-methoxyphenyl-2-propanone	17.8	+	−	−	−
31	1-Methyl-n-vanillyl-2-Phenethamine	17.81	−	−	+	−
32	4-Methoxyphenyl propanoic acid ethyl ester	19.02	+	−	−	−
33	3-(4-Methoxyphenyl) propionic acid ethyl ester	19.02	−	+	−	−
34	Dodecanoic acid ethyl ester	19.26	+	−	−	−
35	Guaiol	19.39	−	+	−	−
36	Eudesmol-Isomere	19.89	−	−	−	−
37	Eudesmol	19.89	−	+	+	−
38	Cardinol	20	−	−	+	−
39	Eudesm-4(14)-en-11-ol	20.15	−	+	+	−
40	alpha-Eudesmol	20.23	−	−	+	−
41	alpha-Bisabolol	20.59	−	+	+	−
42	1-Hydroxy-3-(4-hydroxy-3-methoxyphenyl)2-propanone	20.66	−	−	+	−
43	4-Hydroxy-methoxy-phenyl-2-propenal	20.81	−	−	+	−
44	Benzoic acid benzyl ester	21.43	+	−	+	−
45	Myristic acid ethyl ester	22.16	+	−	−	−
46	Salicylic acid benzyl ester	22.31	+	−	−	−
47	2-Hydroxy-benzoic acid benzyl ester	22.9	−	−	+	−
48	2-Methoxy-benzoic acid benzyl ester	24.29	−	−	+	−
49	Hexadecanoic acid	24.36	−	−	−	+
50	Ethyl hexadecanoate	24.79	−	+	−	−
51	Hexadecanoic acid ethyl ester	24.8	+	−	−	−
52	2,4-Di-tert-butylphenyl benzoate	25.46	−	+	+	−
53	Benzyl cinnamate	25.62	+	+	+	−
54	4-Hydroxy cinnamic acid	25.82	−	+	+	−
55	9-Octadecenoic acid ethal ester	26.86	+	−	−	−
56	Ethyl-9-octadecenoate	26.86	−	+	−	−
57	4-Hydroxy-3-methoxy cinnamic acid	26.98	−	+	+	−
58	Stearic acid ethyl ester	27.2	−	−	−	+
59	4-Hydroxy-methoxy cinnamic acid	27.55	−	+	+	−
60	2′,6′-dihydroxy-4′-methoxy-chalcone	28	+	+	+	+
61	Tricosane	28.57	−	+	−	−
62	Cinnamyl cinnamate	29.21	+	+	−	−
63	4-Acetyl-benzoic acid phenylmethyl ester	29.74	+	−	−	−
64	Benzyl-4-acetylbenzoate	29.74	−	+	+	−
65	Dihydrochrysin	29.84	−	+	+	−
66	Pentacosane	30.68	−	+	−	−
67	4′,5-Dihydroxy-7-methoxy flavone	31.59	−	−	+	−
68	Chrysin	31.93	−	+	+	−
69	Heptacosane	32.64	−	+	−	−
70	Methyl pentacosanoate	33.38	−	+	−	−
71	Nonacosane	34.46	−	+	−	−
HPLC analysis						
1	Caffeic acid	17.26	+	−	+	−
2	*trans-p*-Coumaric acid	19.66	−	−	+	−
3	Pinocembrin	24.42	+	+	+	−
4	Cinnamic acid	26.06	−	−	+	+
5	Galangin	34.41	−	+	+	−

+: Present; −: Absent.

**Table 2:** Antimicrobial activity of EEP and WEP against Gram-positive bacteria.

Microorganisms	ATCC NO.	EEP (mg/mL)						WEP (mg/mL)		Vancomycin (µg/mL)	
		Germany		Ireland		Czech		Germany			
		MIC	MBC	MIC	MBC	MIC	MBC	MIC	MBC	MIC	MBC
*Staphylococcus aureus* Amme	29213	1.2	5	0.3	0.6	0.6	2.5	1.2	5	0.2	1.6
*Staphylococcus aureus* BAA	977	1.2	5	0.6	1.2	0.6	1.2	1.2	5	0.8	1.6
*Staphylococcus saprophyticus*	15305	1.2	>5	0.3	0.6	1.2	2.5	1.2	5	1.6	1.6
*Staphylococcus aureus*	25923	1.2	2.5	0.08	0.1	0.3	0.6	1.2	2.5	0.8	1.6
*Staphylococcus epidermidis*	14990	0.6	1.2	0.6	1.2	0.6	1.2	1.2	5	0.8	3.1
MRSA/NCTC	10442	0.3	0.6	1.2	>5	0.6	1.2	1.2	>5	1.6	1.6
*VRE VanB*	51299	2.5	5	5	>5	2.5	5	2.5	>5	25	>50
*Streptococcus pyogenes*	12344	0.6	1.2	0.08	0.6	0.08	0.1	0.6	2.5	0.4	1.6
*Streptococcus pneumoniae*	49619	0.3	0.6	0.08	0.1	0.08	0.1	0.6	2.5	NT	NT
*Streptococcus oralis*	35037	0.3	0.6	0.1	0.3	0.1	0.3	1.2	5	0.8	0.8
*Streptococcus agalactia*	27956	0.6	1.2	0.1	0.3	0.3	0.6	0.6	5	0.4	0.4
*Streptococcus thermophilus*	19258	0.3	0.6	0.04	0.08	0.08	0.1	0.3	0.6	0.4	0.8
*Bacillus subtilis*	6051	0.3	0.6	0.08	0.3	0.3	0.6	2.5	5	0.4	0.8
*Enterococcus casseliflavus*	70032	2.5	>5	0.6	1.2	1.2	2.5	5	>5	12.5	>50

NT: Not tested.

**Table 3:** Antimicrobial activity of EEP and WEP of propolis against Gram-negative bacteria.

Microorganisms		EEP (mg/mL)						WEP (mg/mL)		Streptomycin (µg/mL)	
	ATCC NO.	Germany		Ireland		Czech		Germany			
		MC	MBC	MIC	MBC	MIC	MBC	MIC	MBC	MIC	MBC
*K. pneumoniae*	700603	5	>5	0.6	>5	1.2	>5	2.5	2.5	1.6	3.1
*Klebsiella pneumoniae* *	800877	>5	NT	>5	NT	>5	NT	1.2	2.5	25	50
*Klebsiella pneumoniae* *	809273	1.2	2.5	0.6	1.2	1.2	2.5	>5	NT	25	50
*Klebsiella oxytoca*	700324	2.5	>5	1.2	>5	2.5	>5	2.5	2.5	3.1	6.2
*Escherichia coli*	25922	5	>5	1.2	>5	0.6	>5	2.5	2.5	3.1	6.2
*Escherichia coli O157:H7*	35150	5	>5	0.6	>5	0.6	>5	1.2	2.5	6.2	12.5
*Pseudomonas aeruginosa*	27853	2.5	>5	0.6	>5	1.2	>5	2.5	5	3.1	12.5
*Salmonella choleraesuis*	554	>5	NT	>5	NT	>5	NT	2.5	5	6.2	25
*Shigella flexneri*	29903	2.5	>5	0.3	>5	0.6	>5	2.5	2.5	3.1	3.1
*Haemophilus influenzae*	49747	2.5	>5	0.6	1.2	1.2	2.5	2.5	5	NT	NT
*Acinetobacter baumannii*	BAAm 747	>5	NT	5	>5	5	5	1.2	0.6	3.1	12.5
*Burkholderia cepacia*	25416	5	>5	1.2	5	1.2	5	1.2	2.5	>50	>50
*Enterobacter cloacae*	700323	>5	NT	>5	NT	>5	NT	2.5	>5	25	50
*Yersinia enterocolitis*	9610	2.5	>5	1.2	5	1.2	5	1.2	2.5	25	25

* Clinical isolate. NT: Not tested.

**Table 4:** Antimicrobial activity of ethanol and water extracts of propolis against fungi.

Microorganisms		EEP (mg/mL)						WEP (mg/mL)		Nystatin (µg/mL)	
	ATCC No.	Germany		Ireland		Czech		Germany			
		MIC	MFC	MIC	MFC	MIC	MFC	MIC	MFC	MIC	MFC
*Candida albicans*	90028	5	>5	0.6	0.6	0.6	1.2	2.5	5	0.2	0.4
*Candida albicans* *	105366	5	>5	0.3	0.3	1.2	2.5	2.5	>5	0.2	0.4
*Candida glabrata* MYA	2950	5	>5	0.3	0.6	0.6	0.6	5	>5	0.2	0.4
*Candida glabrata* *	105410	>5	>5	0.6	1.2	2.5	5	5	>5	6.2	12.5
*Candida glabrata* *	105413	>5	>5	0.1	0.1	0.6	1.2	2.5	2.5	6.2	12.5
*Candida parapsilosis*	22019	1.2	>5	0.3	0.6	0.6	0.6	2.5	5	0.4	0.8
*Candida parapsilosis* *	105328	>5	>5	0.6	0.6	2.5	2.5	2.5	>5	1.3	2.5
*Candida tropicalis*	9968	5	>5	0.2	0.3	0.6	1.2	5	>5	0.8	1.6
*Candida krusei*	90878	>5	>5	0.6	0.6	1.2	2.5	1.2	2.5	25	25

* Clinical isolated.

**Table 5:** Results of the checkerboard assay with fractional inhibitory concentration and FIC indices of two-drug combinations between propolis extract (EEP) and antibiotics.

Microorganisms	ATCC NO.	Agent	MIC (µg/mL)		FIC	FICI	Interpretation
			Alone	Combination			
MRSA	10442	EEP	600	150	0.3	0.38	Synergy
		Vancomycin	1.6	0.2	0.1		
		EEP	300	75	0.3	0.5	
		Oxacillin	50	12.5	0.3		
*E. faecalis*	51299	EEP	2500	312.5	0.1	0.4	Synergy
		Vancomycin	25	6.25	0.3		
*S. pneumoniae*	49619	EEP	80	20	0.3	0.5	Synergy
		Levofloxacin	0.4	0.1	0.3		
*H. influenza*	49747	EEP	600	150	0.3	0.5	Synergy
		Levofloxacin	0.008	0.002	0.3		
*S. pyogenes*	12344	EEP	80	20	0.3	0.5	Synergy
		Vancomycin	0.4	0.1	0.3		
